# Automated Identification of Ventricular Tachycardia Ablation Targets: Multicenter Validation and Workflow Characterization

**DOI:** 10.19102/icrm.2022.130903

**Published:** 2022-09-15

**Authors:** Ahmed Niri, Einat Shapira, Stéphane Massé, Meir Bar-Tal, Tal Bar-on, Gal Hayam, Amir Ben-Dor, Abishek Bhaskaran, Andrew Ha, Elad Anter, Andreu Porta-Sanchez, Nicholas Jackson, Kumaraswamy Nanthakumar, Krishnakumar Nair

**Affiliations:** ^1^The Hull Family Cardiac Fibrillation Management Laboratory, Peter Munk Cardiac Centre, Toronto General Hospital, University Health Network, Toronto, Ontario, Canada; ^2^Biosense Webster, Haifa, Israel; ^3^Department of Cardiovascular Medicine, Heart and Vascular Institute, Cleveland Clinic, Cleveland, USA; ^4^Arrhythmia Unit, Hospital Clinic de Barcelona, Barcelona, Spain; ^5^John Hunter Hospital, Newcastle, New South Wales, Australia

**Keywords:** Cardiac mapping, substrate mapping, VT ablation

## Abstract

Decrement evoked potentials (EPs) (DeEPs) constitute an accepted method to identify physiological ventricular tachycardia (VT) ablation targets without inducing VT. The feasibility of automated software (SW) in the detection of arrhythmogenic VT substrate has been documented. However, multicenter validation of automated SW and workflow has yet to be characterized. The objective of this study was to describe the functionality of a novel DeEP SW (Biosense Webster, Diamond Bar, CA, USA) and evaluate the independent performance of the automated algorithm using multicenter data. VT ablation cases were performed in the catheterization laboratory and retrospectively analyzed using the DeEP SW. The algorithm indicated and mapped DeEPs by first identifying capture in surface electrocardiograms (ECGs). Once capture was confirmed, the EPs of S1 paces were detected. The algorithm checked for the stability of S1 EPs by comparing the last 3 of the 8 morphologies and attributing standard deviation values. The extra-stimulus EP was then detected by comparing it to the S1 EP. Once detected, the DeEP value was computed from the extra-stimulus and displayed as a sphere on a voltage map. A total of 5,885 DeEP signals were extracted from 21 substrate mapping cases conducted at 3 different centers (in Spain, Canada, and Australia). A gold standard was established from ECGs manually marked by subject experts. Once the algorithm was deployed, 91.6% of S2 algorithm markings coincided with the gold standard, 1.9% were false-positives, and 0.1% were false-negatives. Also, 6.4% were non-specific DeEP detections. In conclusion, the automated DeEP algorithm identifies and displays DeEP points, revealing VT substrates in a multicenter validation study. The automation of identification and mapping display is expected to improve efficiency.

## Introduction

Ventricular tachycardia (VT) ablation is an effective therapy, even for patients failed by anti-arrhythmic medications.^1^ In a previous publication, we demonstrated that VT ablation leads to a decrease in health care use.^2,3^ The optimal approach for VT ablation is not known. Presently, multiple VT mapping methods/strategies—such as activation and substrate mapping—are employed to identify putative target sites. However, the use of activation mapping is limited due to hemodynamic instability during VTs or the poor inducibility of VT. For these reasons, substrate-based ablation is commonly performed with an alternative strategy to identify diastolic channels that participate in the maintenance of the re-entrant circuit.

We have developed decrement evoked potential (EP) (DeEP) mapping as a novel method shown to be an effective, focused, and minimalistic strategy to identify arrhythmogenic sites without the need to induce VT. This approach was initially developed from human intraoperative data,^2^ which were tested in a computer-modeled simulation^4^ and then prospectively validated in a randomized multicenter trial.^3^ This methodology has been included in recent Heart Rhythm Society/European Heart Rhythm Association/Asia Pacific Heart Rhythm Society/Latin-American Heart Rhythm Society VT guidelines for substrate-guided VT ablation.^5^

During VT substrate mapping, DeEPs are traditionally identified and measured after a successful pacing train with short extra-stimuli. To date, manually identifying DeEPs is the only known method to create maps in order to pinpoint target zones. To simplify the process of identifying arrhythmogenic myocardium that maintains VT, a novel automatic software (SW) was developed for DeEP detection and display. The impetus for this project was an objective, non–operator-dependent detection of DeEPs that is automated and efficient to operate in near–real time. This automatic SW determines capture, tracks pacing and myocardial EPs in order to detect the decrements, and populates and displays DeEP maps, identifying VT targets. Here, we detail the performance of the novel automated algorithm.

## Methods—work flow

This study conforms to the Helsinki principle for clinical studies and was approved by the institutional research ethics committee of the University Health Network.

The cases used in this study were all VT ablation cases performed in 3 different medical centers across the world—located in Canada, Spain, and Australia. All centers used the previously defined DeEP procedure to identify and ablate arrhythmogenic myocardium. After the completion of each catheter laboratory case, data were extracted from the CARTO^®^ 3 system (Biosense Webster, Diamond Bar, CA, USA), and signals were retrospectively analyzed using the DeEP automatic SW (Biosense Webster). Once analyzed, 2 electrophysiology experts revised the DeEP markings of the automatic SW. Descriptive statistics—in the form of percentages—were used to highlight the outcome of the DeEP SW performance. SW performance was also measured in the form of true-positive (TP), true-negative (TN), false-positive (FP), and false-negative (FN) values. A TP result indicated that both the expert and the SW agree that there is DeEPs. A TN result indicated that both the expert and the SW agree that there is no DeEPs. An FP result indicated that the SW has found DeEPs, whereas the expert has found none. Finally, a FN result indicated that the SW did not find any DeEP but the expert found DeEPs. Accordingly, sensitivity and specificity were calculated as follows:


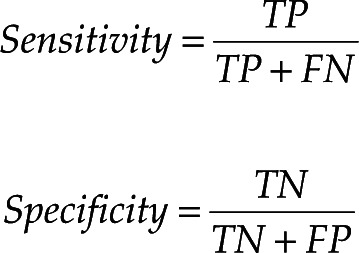


Cohen’s kappa value was also used to quantify the agreement between experts. The Cohen kappa calculation was performed as follows:


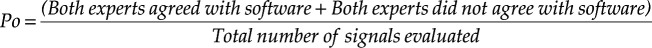


## Decrement evoked potential procedure

**Figure 1:**
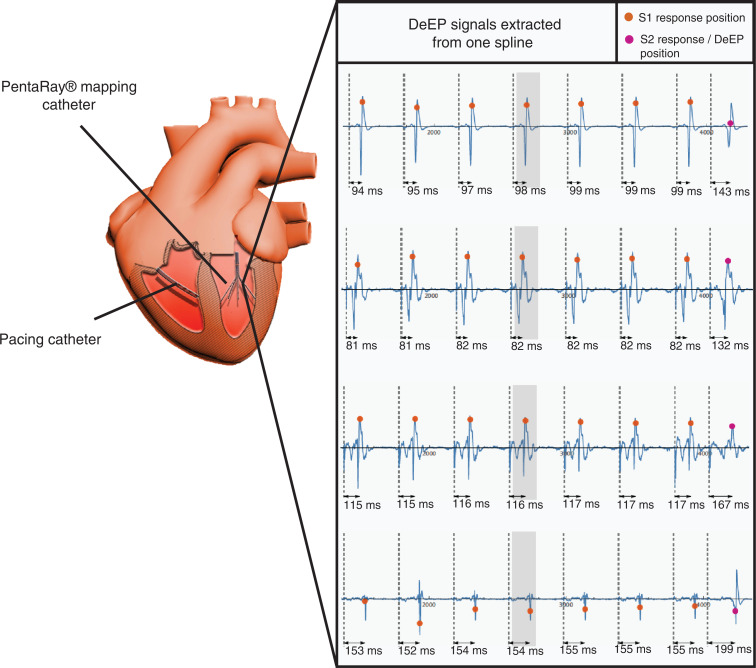
Display of the position of the catheters during catheter laboratory procedures. **Left:** A pacing catheter is placed in the right ventricular apex and a PentaRay^®^ (Biosense Webster) catheter is mapping the left ventricle. **Right:** From the PentaRay^®^ catheter, electrograms extracted from 1 spline are shown. The signals display the pace train and the extra-stimulus derived to extract the decrement evoked potentials. *Abbreviation*: DeEP, decrement evoked potential.

During each case, a standardized DeEP procedure was performed to help develop and ensure the precision of the automatic SW. Once the catheter was positioned in the desired location, the operators delivered a pace train (S1) with a cycle length of 600 ms from the right ventricle. One or more extra-stimuli were then delivered—at the ventricular effective refractory period **(Figure 1)**—to identify isolated near-field delayed potentials. Based on the delay produced by the extra-stimulus, the arrhythmogenic myocardium was identified as the area of interest for ablation therapy.

## Decrement evoked potential software automatic detection

Once the procedure was completed, the data gathered during the case were extracted from the CARTO^®^ 3 system and intentionally saved as an “error report.” The approach provided us with all the details of the case, including signals, maps, and ablation locations. In order to develop and test the automatic DeEP SW, the signals extracted and the 3-dimensional (3D) coordinates of the cardiac chamber were the main data used.

The SW built consists of 5 main functions: (1) pace detection, (2) capture confirmation, (3) S1 EP detection and detection of extra-stimulus EP, (4) extraction of DeEP value, and (5) 3D mapping of DeEP values. These functions are explained in more detail below.

### Pace detection

By rerouting the electrical stimulation into the CARTO^®^ 3 system, the algorithm was able to identify the pacing artifact. The pace detection section of the algorithm starts by identifying the S1 pacing morphologies using the peaks of the pacing artifacts. The extra-stimulus is then detected—this stage confirms the existence or the absence of S2. If the extra-stimulus is not found, the algorithm will terminate, and no DeEP calculation will be performed. If the presence of S2 is confirmed, the algorithm for DeEP detection will continue.

### Capture confirmation

**Figure 2:**
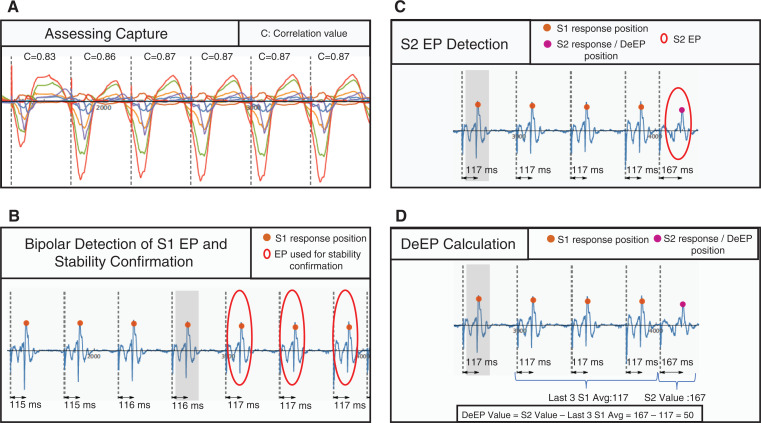
Workflow of the decrement evoked potentials (EPs) automatic software. **A:** The software first assesses capture by associating correlation values with the surface electrocardiogram. **B:** The last 3 bipolar EPs of the pacing train are then detected and stability is confirmed. **C:** The S2 EP is detected. **D:** Finally, the decrement EPs value is then calculated by subtracting the last S1 detection time delay from the S2 detection time delay. *Abbreviations*: DeEP, decrement evoked potential; EP, evoked potential.

After completing the pace detection and confirming the delivery of an extra-stimulus, the automatic DeEP algorithm attempts to confirm capture by calculating a correlation value of the signals for each segment of the surface electrocardiogram’s EPs, as seen in **Figure 2A**.

### S1 evoked potential detection and detection of the extra-stimulus

Based on the capture confirmation and S1 detection, the automated SW then attempts to detect the extra-stimulus and calculates and extracts a DeEP value. As can be observed in **Figures 1 and 2**, the extra-stimulus is delivered prematurely in order to stress the substrate. Due to the change in the pacing stimulus interval, the DeEP SW detects the extra-stimulus. If the detected extra-stimulus is registered to be >10% smaller than the pacing train interval, the SW will cease the detection.

### Extraction of the DeEP value

Once the detection of the extra-stimulus is confirmed, the S2 EP is detected and compared to the S1 EPs in order to calculate the decremented value. The automatic SW calculates the DeEP by subtracting the time delay of the EP of the extra-stimulus from the time delay of the latest S1 EP—as shown in **Figures 2C and 2D**. This difference in time delay results in a DeEP value calculated for each bipolar electrode along the catheterized probe.

### Mapping of DeEPs

Once the DeEP values have been calculated, the 3D anatomical map of the cardiac chamber is extracted from the error log and a 3D voltage map is built. The SW places projected DeEP spheres on the voltage map—each sphere size corresponds to the DeEP value calculated. A minimum DeEP value threshold can be defined by the user to limit the display to decrements of interest, typically >10 ms, as seen in **Figure 5**.

## Results

### Performance

In a total of 21 cases in 3 different centers, 5,885 DeEP signals were extracted from the CARTO^®^ 3 export files. Following data extraction from each case, the algorithm processed the electrograms to automatically mark the EPs and compute DeEP values. To evaluate the performance of the algorithm, subject experts evaluated the markings and manually corrected them as they deemed appropriate, yielding a gold standard. The performance of the algorithm was assessed based on the following 4 criteria:

1. Unchanged detections: Detections by the algorithm that were deemed appropriate by subject experts and remained unchanged during manual correction
2. Changed detections: Detections by the algorithm that were deemed questionable and consequently revised and manually corrected by subject experts
3. Deleted detections: Detections by the algorithm that were deemed inappropriate and consequently removed by subject experts
4. Added detections: Detections made by subject experts that the algorithm had missed

**Figure 3:**
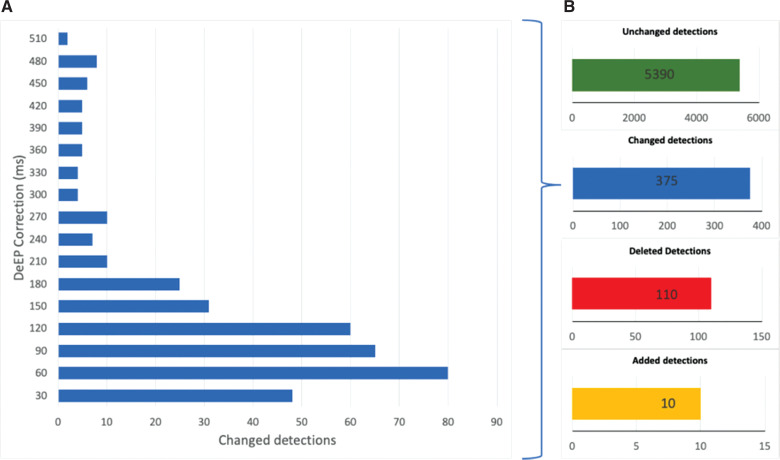
Bar graphs demonstrating the performance of the automatic decrement evoked potentials algorithm. On the left side is a detailed bar graph demonstrating the number of changed detections and their corrected decrement evoked potential values. On the right side are the parameters that assess the performance of the algorithm—namely, unchanged detections, changed detections, deleted detections, and added detections. *Abbreviation*: DeEP, decrement evoked potential.

Overall, of the 5,885 DeEP detections made by the algorithm and the subject experts, 91.6% (n = 5,390) were unchanged detections, 6.4% (n = 375) were changed detections, 1.9% (n = 110) were deleted detections, and 0.2% (n = 10) were added detections. This is depicted in **Figure 3B**. For all corrections made, **Figure 3A** shows the volume of corrections at different time intervals. It is apparent that there was a greater number of signals corrected at lower DeEP timings. The corrections dramatically decreased as the DeEP timing increased to >150 ms.

**Figure 4:**
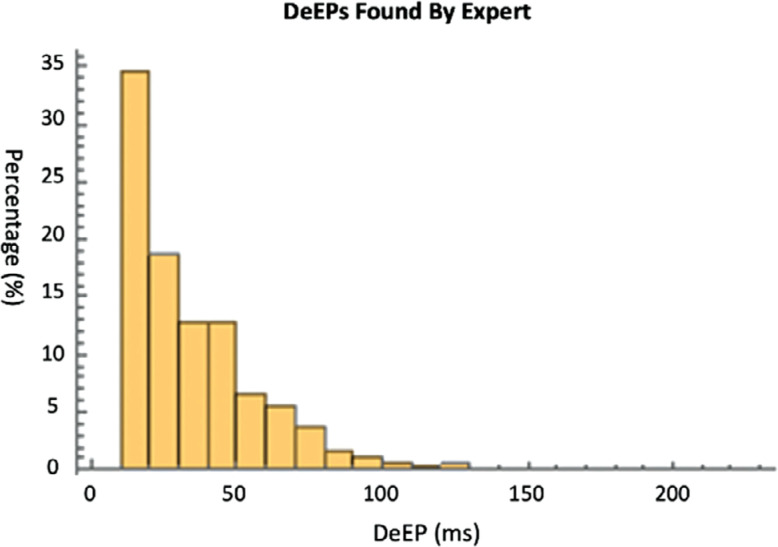
Bar graphs demonstrating the decrement evoked potentials (DeEPs) found by experts. This displays all DeEPs (true-positive + false-negative) where more smaller DeEPs are identified. *Abbreviation*: DeEP, decrement evoked potential.

In order to reduce bias by the different terminal screens and viewers, we had the reviewers use the same terminal/same viewer to annotate the signal to produce the most robust comparison. This analysis performed using the same viewer/terminal to compute agreement between the 2 experts was conducted on a subset of signals (3,860 signals). Out of these 3,860 signals, the first expert identified 1,451 TPs, 1,812 TNs, 136 FPs, and 281 FNs—with an 83.8% sensitivity and a 93% specificity. Compared to the DeEP algorithm, the first expert had a similar marking 80.2% of the time. The second expert identified 1,562 TPs, 2,016 TNs, 25 FPs, and 77 FNs—with a 95.3% sensitivity and a 98.8% specificity. Compared to the DeEP algorithm, the second expert had a similar marking 97.7% of the time. The majority of DeEPs that were found by the experts were seen on the lower end of DeEP timings (<50 ms), as seen in **Figure 4**. The calculated Cohen kappa showed that the 2 experts had 88% similarity in their markings.

**Figure 5:**
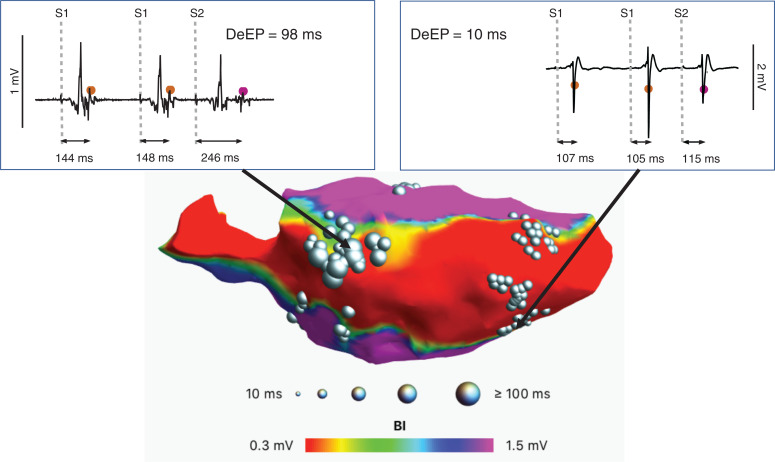
Display of decrement evoked potential points positioned over a voltage map. The size of the spheres indicates the amount of decrement from the signals. Above the map, 2 signals were chosen on the map—representing differently sized spheres—to demonstrate the difference in decrement evoked potential location and identification. *Abbreviation*: DeEP, decrement evoked potential.

**Figure 5** displays a resulting voltage map overlaid with the algorithm’s accepted DeEP points. As shown in previous studies, these points clearly co-localize with voltage boundaries and guide the operator toward possible ablation sites.

## Conclusion

The DeEP concept identifies critical components of the VT circuit without having the need to induce VT. We have developed a completely automated algorithm and workflow for use in the catheter laboratory to identify VT ablation targets based on DeEPs. A critical evaluation of the algorithm suggests that the performance of the algorithm has reached a confidence level for deploying the algorithm for DeEP detection and VT circuit identification during clinical cases. Once the algorithm is deployed, the metrics of automatic detection and mapping display of DeEP efficiency will be determined.
